# Definition and Classification of Generic Drugs Across the World

**DOI:** 10.1007/s40258-014-0146-1

**Published:** 2015-06-20

**Authors:** Rafael Alfonso-Cristancho, Tatiana Andia, Tatiana Barbosa, Jonathan H. Watanabe

**Affiliations:** SORCE, Department of Surgery, School of Medicine, University of Washington, 1107 NE 45th Street, Suite 502, Seattle, WA 98105 USA; Brown University, Providence, USA; RANDOM Foundation, Bogota, Colombia; University of California San Diego Skaggs School of Pharmacy and Pharmaceutical Sciences, La Jolla, CA USA

## Abstract

Our aim was to systematically identify and compare how generic medications, as defined by the US Food and Drug Administration (FDA), World Health Organization (WHO), and European Medicines Agency (EMA), are classified and defined by regulatory agencies around the world. We focused on emerging markets and selected the most populated countries in each of the WHO regions: Africa, the Americas, Eastern Mediterranean, Europe, Southeast Asia, and Western Pacific. A structured review of published literature was performed through December 2013. Direct information from regulatory agencies and Ministries of Health for each country was extracted. Additionally, key informant interviews were performed for validation. Of the 21 countries selected, approximately half provided an official country-level definition for generic pharmaceuticals. The others did not have any definition or referred to the WHO. Only two-thirds of the countries had specific requirements for generic pharmaceuticals, often associated with clinical interchangeability. Most countries with requirements mention bioequivalence, but few required bioavailability studies explicitly. Over 30 % of the countries had other terms associated with generics in their definitions and processes. In countries with generic drug policies, there is reference to patent and/or data protection during the drug registration process. Several countries do not mention good manufacturing practices as part of the evaluation process. Countries in Africa and Eastern Mediterranean regions appear to have a less developed regulatory framework. In summary, there is significant variability in the definition and classification of generic drugs in emerging markets. Standardization of the definitions is necessary to make international comparisons viable.

## Key Points for Decision Makers

**Table:** 

The classification and definition of generic pharmaceutical products is different across the world.
The impact of these differences in the definition and requirements of generic pharmaceutical products is unknown.
The differences in the definition and classification of generic pharmaceutical products must be taken into consideration when performing international comparisons, including the impact of drug policies.

## Background

The World Health Organization (WHO), during the World Health Assembly in 1975, published a resolution to develop means to assist Member States in formulating national drug policies [1]. Since then, and following the recommendations of the WHO, many countries have developed their own national drug policies [2]. The framework of these recommendations is often considered to be built around improving access to “essential drugs” that in most cases mirror the *Essential Medicines List* from the WHO [3], currently in its 18th edition [4]. According to the WHO, this list includes the most efficacious, safe and cost-effective medicines for priority conditions. Most of the drugs included are off-patent and available as generic products, which are often offered at lower prices than the innovator branded product, potentially reducing costs for patients and the healthcare system [5]. The use of generic pharmaceutical products is then promoted, in order to reduce costs and increase access to healthcare [6]. But, despite highlighting the need for rigorous quality and safety assessments for pharmaceutical products in order to achieve these goals, the quality of pharmaceutical products available in the market in many developing countries varies, in part because of the lack of clear and specific requirements for generic pharmaceutical products [7, 8].

Currently, the use of generic pharmaceutical products represents over half of the total volume of pharmaceutical products used worldwide but only 18 % of the total value of the pharmaceutical market [9]. These proportions vary by region and country, but the consumption of generic pharmaceutical products is consistently higher than that of innovators in most countries, being one of the most used healthcare technologies around the world [10]. The WHO defines a generic product as “a pharmaceutical product, usually intended to be interchangeable with an innovator product, that is manufactured without a license from the innovator company and marketed after the expiry date of the patent or other exclusive rights” [11]. In the USA, the Food and Drug Administration (FDA) has stated that, “A generic drug is identical—or bioequivalent—to a brand name drug in dosage form, safety, strength, route of administration, quality, performance characteristics and intended use” [12]. Finally, the European Medicines Agency (EMA), the main regulatory body for pharmaceutical products in the EU, defines a generic medicinal product as a “product which has the same qualitative and quantitative composition in active substances and the same pharmaceutical form as the reference medicinal product, and whose bioequivalence with the reference medicinal product has been demonstrated by appropriate bioavailability studies. (Reg. 726/2004, Art 10, 2b)” [13].

These definitions are critical when regulatory agencies in each country determine the requirements and standards that pharmaceutical products must follow in order to obtain approval and reach the market. Minor differences in wording may have a great impact on how these products are assessed and the standards that must be followed. For example, using words such as “interchangeable”, “identical” or “bioequivalent”, which are used by the WHO, FDA and EMA, respectively, have important connotations with regard to determining the evidence required from a manufacturer of generic products in order for them to obtain approval by regulatory agencies and reach the market in specific countries. The concepts of bioequivalence and interchangeability are of particular importance in these definitions. In theory, a generic drug is considered interchangeable with an innovator or a reference pharmaceutical product when there is evidence demonstrating that it can be as effective and safe for patients in the specific indication. That evidence is often but not always obtained through bioavailability and bioequivalence studies comparing the generic to the innovator as the reference product.

If the definition of generic drug in the regulation of pharmaceutical products in a specific country involves the terms “interchangeable” or “bioequivalence”, as previously described, it will generally increase the supporting evidence required from manufacturers when submitting a new generic application. On the other hand, the absence of these terms from the definition may be interpreted as if that evidence is not required or is required only for specific high-risk drugs, allowing for products without demonstrated “interchangeability” or “bioequivalence” to be approved and brought onto the market.

Reductions in national drug spending of more than 40 % have been estimated if generic penetration reached a maximum in each country [14]. In the USA, generic medications cost less than one-third of their branded counterparts [15]. The reduced price allows providers to treat more patients effectively with the same amount of overall dollars. However, these concepts are conditional on bioequivalent generics being substituted. While numerous cost-savings analyses have been conducted suggesting significant reductions in drug expenditure, these analyses have been conducted in settings where bioequivalent generics were regulated and prevalent. In developing countries, we are currently limited to conjecturing possible cost savings from appropriate generic substitution. This manuscript sheds light on the likelihood that a generic medication is a clinically appropriate substitute.

The definition and classification of generic pharmaceutical products are not the same across the world. Our aim is to describe and compare the differences in the classification and definitions of generic pharmaceutical products in the largest developing countries, where more than 80 % of the world’s population is currently living and receiving healthcare [16].

## Methods

**Fig. 1:**
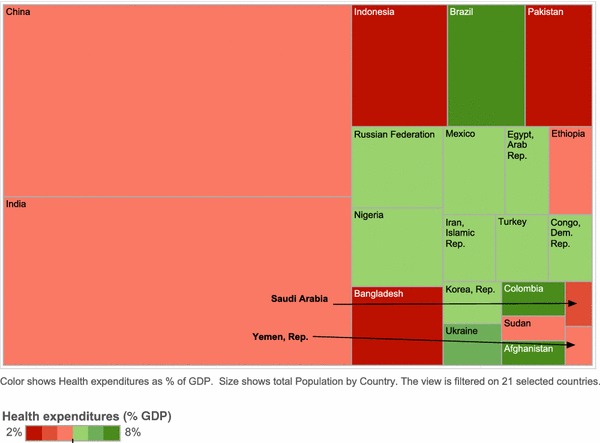
Population and healthcare expenditure as percentage of GDP. *GDP* gross domestic product

Using the six WHO geographic regions (the Americas, Africa, Southeast Asia, Europe, Eastern Mediterranean, Western Pacific), we identified the developing countries with the largest population in each region and selected a total of 21 countries for the analysis: Brazil, Mexico, Colombia, Nigeria, Ethiopia, Democratic Republic of Congo, India, Indonesia, Bangladesh, Russia, Turkey, Ukraine, Pakistan, Egypt, Iran, Sudan, Saudi Arabia, Afghanistan, Yemen, China and Korea (Table 1). Additional economic information about gross domestic product (GDP) per capita and the percentage of GDP devoted to healthcare expenditures was also extracted as a proxy for the weight given to healthcare investment as public policy (Fig. 1).

**Table 1:** Characteristics of countries selected by WHO region

Country	Population	Health expenditures as % of GDP
China	1,302,350,455	4.0
India	1,139,737,707	4.0
Indonesia	226,999,190	2.0
Brazil	185,416,160	7.0
Pakistan	158,874,699	2.0
Russian Federation	143,593,754	5.0
Nigeria	140,307,544	4.0
Bangladesh	139,913,660	3.0
Mexico	106,602,288	5.0
Egypt, Arab Rep.	74,266,212	5.0
Ethiopia	74,253,849	4.0
Iran, Islamic Rep.	69,724,855	5.0
Turkey	68,174,186	5.0
Congo, Dem. Rep.	57,535,109	4.0
Korea, Rep.	48,230,364	5.0
Ukraine	47,264,656	6.0
Colombia	43,038,783	6.0
Sudan	30,655,713	3.0
Afghanistan	30,005,286	7.0
Saudi Arabia	23,864,238	3.0
Yemen, Rep.	20,741,624	4.0
Total population selected countries	4,131,550,331	4.4

*Dem.* Democratic, *GDP* gross domestic product, *Rep.* Republic, *WHO* World Health Organization

An individual country profile was created using information from published literature, available through PubMed and Google Scholar, and official government sources available online. Websites from the Ministry of Health, regulatory agencies or other government sites were identified for each of the selected countries and reviewed to extract the most recent and relevant information about the definition and classification of pharmaceutical products, focusing on generic pharmaceutical products. Finally, key informant interviews were performed with experts in regulation of pharmaceutical products for specific countries where information was limited or where additional confirmation was required to complete the information.

The information was summarized and standardized for comparison across countries, focusing on the main components of the definitions and classification of pharmaceutical products (until December 2013).

## Results

The information available about the regulation and definition of generic pharmaceutical products in each of the 21 countries included in the analysis was very heterogeneous, with different levels of access to the information. Some countries had information that was easy to access and review about the regulation and definition of pharmaceutical products, but many others did not. Of the 21 countries selected, we were able to identify specific information about the definition of generic pharmaceutical products from government or other official sources from only 13 countries (62 %). For five countries (24 %) we located generic medication related-details, but not from “official” government sources. For Afghanistan, the information was under review by the WHO. For Yemen and Colombia, we could not identify specific definitions for generic pharmaceutical products via government or other official sources^1^ (Table 2).

Interestingly enough, in 20 of the 21 countries selected, we were able to identify references to country-specific pharmaceutical drug policies, mostly in concordance with the initial resolution of the WHO in the late 1970s and its updates since then. Consistently, these same countries have references to most of the components of the pharmaceutical policy delineated by the WHO, which includes selection of essential medicines, affordability, drug financing, supply system, drug regulation, research, and monitoring and evaluation. The only country without an explicit reference to a country-specific pharmaceutical drug policy was Korea, which, in fact, is notably absent from the WHO’s list of countries with essential medicines lists [15]. Nevertheless, Korea’s regulatory policies mention other important components of the WHO pharmaceutical policy framework, focusing on drug financing, drug regulation, and monitoring and evaluation.

In almost a third of the countries, where information was available, there are other terms associated with generic products, making market differentiation difficult. Those terms are sometimes based on whether or not the generic product is branded, or if it has been labeled as interchangeable, or as a similar product. Usually, “similar” pharmaceutical products, sometimes called “copies”, are pharmaceutical products with the same International Nonproprietary Name (INN) as the reference generic pharmaceutical product, but with limited or no evidence of bioequivalence and bioavailability. Most of the countries selected in this study (16 of 21), mentioned requiring some level of bioequivalence testing, mostly in vitro, to provide a specific label for generic pharmaceutical products.

A requirement to have the innovator pharmaceutical product off-patent in order to have a generic approved seems to be consistently reported across all countries that are in line with the Intellectual Property Rights Related to Trade (TRIPS) agreement, which requires all World Trade Organization members to adhere to it [16]. Nevertheless, the fact that many pharmaceutical companies decide not to file patents in every country allows for generic pharmaceutical products to emerge in those countries despite the product being under patent in other countries.

Another important consideration for the quality of generic pharmaceutical products is the requirement of good manufacturing practices (GMP); except for some countries in the Middle East, all countries included in the analysis had some documentation referring to GMP as part of the local pharmaceutical policy. Most countries did not explicitly mention tax exemptions or incentives for production or importation of generic pharmaceutical products. In the same token, most countries did not offer specific market protection for generic products.

## Discussion

Using selected developing countries from every WHO region, on the basis of population size, we examined the current pharmaceutical policies and regulation focusing on the definition and requirements for generic pharmaceutical products. We found important differences between countries in terms of the definitions used for pharmaceutical generic products and other terms or definitions associated with generic products, such as similars, copies, branded generic products, etc. These differences and inconsistencies bring important challenges for international comparisons that must be addressed and recognized. The availability of generic products with different standards in each country limits the generalizability of the assessment of country-specific policies, as well as the pooling of information from multiple countries to assess the impact of generic pharmaceutical products on general or specific health outcomes. Previous research has recognized these differences in specific countries or regions [17, 18]. In 2011, Vacca et al. [19] published a report comparing the pharmaceutical regulation regarding generic products in 14 countries in the Americas, showing three levels of policy for generic pharmaceutical products: (1) countries with minimal or no specific regulation; (2) countries with regulation, but without restrictions for substitutions between different types of generics; and (3) countries with specific regulation and restrictions if therapeutic equivalence is not demonstrated [20]. If we applied a similar framework to our analysis, most of the countries in our sample would be grouped in levels 2 and 3. Vacca et al. placed most of the countries in level 1, minimal or no regulation. However, the countries included in their selection were largely countries with less developed economies and relatively smaller populations than those we included for review here. Our analysis sought to capture a wider representation of countries in terms of population size and economic scale. This likely explains the differences in relative proportions found in our study compared with their work.

In 2013, Nguyen et al. [21] performed a summary description of generic medicines policies in Asia Pacific. The authors, who retrieve the information from a Workshop in the region performed in 2012, said that many countries in this region did not have a generic medicines policy within their national medicines policy, and that only a few countries had comprehensively implemented generic medicines policies with strong regulatory requirements. The participants on the workshop identified barriers to successful implementation of generic medicines policies, including mistrust of the quality of generic pharmaceutical products and the lack of inspectors or regulatory bodies to assess them properly. Finally, it was reported that the financial benefit from generic substitution had not been measured or was unclear in many countries, despite clear incentives to implement it. Our results are in line with the initial points made by the authors. The local perspectives about generic policies were out of our scope since we focused on information published or available from official sources and not from surveys or workshops.

Vogler [22], in 2012, performed an analysis on the impact of pharmaceutical pricing and reimbursement policies on generics uptake in 29 European countries. Although the focus of the paper was on reimbursement of generics, the author highlights some differences between countries in the definition and use of generics in these countries.

Clearly, as expressed in the previous reports, one of the main challenges for this type of international analysis is the heterogeneity in the availability and structure of the information about pharmaceutical policies and regulations in each country. Continuous changes in the healthcare system and changes in regulatory processes and policies for the assessment and coverage of health technologies, including pharmaceutical products, in each country are also difficult to follow closely and understand regarding the potential impact across regions.

**Table 2:** Generic medication regulatory features by country

				Pharmaceutical policy components							Establishes therapeutic equivalence studies					Economic incentives	
Country	Generic definition (Y/N)	Reference to international standards in the definition	Generic drugs policy	Selection of essential drugs	Affordability	Drug financing	Supply system	Drug regulation	Research	Monitoring and evaluation	Bioequivalence mandatory, selective or not required	In vitro tests	Off-patent	Regulatory ethical aspects	Fulfillment of good manufacturing practices	National tax exemption	Promotes generics production
Brazil	Y	Y	Y	Y	Y	Y	Y	Y	Y	Y	Y	Y	Y^b^	Y	Y	NA	Y
Colombia	N		Y	Y	Y	Y	Y	Y	Y	Y	N	N	Y^b^	Y	Y	N	NA
México	Y	N	Y	Y	NA	Y	Y	Y	Y	Y	N	Y	Y^b^	Y	Y	NA	NA
Nigeria	Y	N	Y	Y	Partial	Y	PS	Y	Y	Y	Y	Y	Y^b^	Y	Y	Y	NA
Ethiopia	Y	N	Y	Y	NA	Y	P&PS	Y	Y	Y	Y	Y	Y	Y	Y	NA	NA
Democratic Republic of Congo	Y	N	Y	Y	Y	NA	NA	Y	Y	Y	Y	NA	Y^b^	NA	Y	NA	NA
India	Y	N	Y	Y	NA	Partial	NA	Y	Y	Y	Y	Y	Y^b^	Y	Y	NA	Y
Indonesia	Y	N	Y	Y	N	Partial (for PH)	DHS	DHS	Y	Y	N	NA	Y^b^	Y	Y	NA	Y
Bangladesh	Y^a^	Y	Y	Y	Y	NA	P&PS	Y	Y	Y	N	N	Y^b^	NA	Y	NA	NA
Russian Federation	Y^a^	Y	Y	Y	NA	Y	Y	Y	NA	NA	Y	Y	Y^b^	Y	Y	NA	Y
Turkey	Y	N	Y	Y	Y	Y	NA	NA	Y	Y	Y	Y	Y^b^	Y	Y	NA	NA
Ukraine	Y^a^	Y	Y	Y	NA	NA	P&PS	Y	Y	Y	Y	NA	Y^b^	Y	Y	NA	NA
Pakistan	Y	N	Y	Y	Partial	Y	NA	Y	Y	Y	Y	N	Y^b^	NA	Y	NA	Y
Egypt	Y	N	Y	Y	Partial	Y	P&PS	Y	Y	Y	Y	NA	Y	Y	NA	NA	Y
Iran	Y^a^	Y	Y	Y	Y	Y	P&PS	Y	Y	Y	Y	NA	Y	NA	NA	Y	Y
Sudan	Y^a^	Y	Y	Y	Y	Y	PH	Y	Y	Y	Y	NA	Y	NA	Y	^c^	NA
Saudi Arabia	Y	N	Y	Y	NA	Y	P&PS	Y	Y	Y	Y	NA	Y	Y	NA	NA	Y
Afghanistan	N (in review by WHO)	N	Y	Y	NA	Y	Y	Y	Y	Y	Y	N	Y	Y	NA	NA	NA
Yemen	N		Y	Y	NA	Y	P&PS	Y	Y	Y	Y	NA	Y	Y	Y	NA	NA
China	Y^a^	Y	Y	Y	Partial	Y	PS	Y	Y	Y	Y	N	Y	Y	Y	^c^	Y
Korea	Y	Y	NA		Y	Y	P&PS	Y	Y	Y	Y	NA	Y	Y	Y	NA	Y

*GMP* Good manufacturing practices, *DHS* decentralized health system, *N* no, *NA* not applicable, *P&PS* private and public sector, *PH* public health, *WHO* World Health Organization, *WTO* World Trade Organization, *Y* yes

^a^This information corresponds to that found in unofficial sources

^b^According to the WTO in 1995, included the ‘Agreement on Intellectual Property Rights Related to Trade’ (TRIPS Agreement), which requires WTO member countries patent protection for at least 20 years from the date of submission of all new technologies, including pharmaceuticals. In some cases, allows members to authorize use by third parties (compulsory licensing) or for non-commercial public (government) use without permission of the patent holder

^c^For these cases, some companies provide GMP through selection of institutions, management of product distribution, and applications for tax exemption

On the extreme end of medication production is drug counterfeiting: the creation of medications that claim to have an active ingredient when they are devoid of the chemical. As more of the world’s medicine production has shifted to the developing world, recent studies have attempted to investigate this phenomenon. The WHO speculated that up to 30 % of the total drug market in developing countries without regulatory oversight may be counterfeit. However, the WHO used a broad definition of counterfeit drugs that included mislabeled or fraudulently labeled drugs even if they contained the correct active ingredients [22]. The challenge is not a simple matter of reducing the supply of counterfeit drugs; it will also entail reducing consumer demand. In some developing countries, patients or their caregivers actively seek cheap medications, supplied without a preceding prescription, from non-licensed peddlers. These demand-side forces have incentivized a counterfeit medication para-economy. The WHO has recommended that community-based organizations disseminate information to improve surveillance of drug counterfeiters and for governmental and non-governmental education campaigns to reduce consumption of counterfeit medications. This again potentiates the need for clear development of a patient-centered definition of a generic medication.

## Conclusions

In summary, the significant variability in the definition and classification of generic pharmaceutical products in emerging and developing countries limits the opportunity to compare and analyze the impact of policies and programs that incentivize their use. Standardization of the definitions is necessary to make international comparisons viable. Global efforts are underway to harmonize the definition and regulation of generic medicines. However, these attempts will likely need to be accelerated given the increased availability of medications that are past patent expiry.

## Footnotes

1 However, in the case of Colombia, the regulation makes explicit reference to the notion of “bioavailability,” suggesting an explicit decision to avoid distinctions between originator and generic drugs (Decreto 677 de 1995).

## References

[1] World Health Organization. How to develop and implement a national drug policy. World Health Organization; 2001.

[2] Laing R, Waning B, Gray A, Ford N, t Hoen E (2003). 25 years of the WHO essential medicines lists: progress and challenges. Lancet.

[3] Attridge CJ, Alexander SP. Improving access to medicines in developing countries. Washington, DC: The World Bank; 2005.

[4] World Health Organization. WHO model list of essential medicines: 18th list. 2013.

[5] Opderbeck DW (2005). Patents, essential medicines, and the innovation game. Vand L Rev.

[6] World Health Organization (2001). How to develop and implement a national drug policy.

[7] Nardi R, Masina M, Cioni G, Leandri P, Zuccheri P (2014). Generic-equivalent drugs use in internal and general medicine patients: distrust, confusion, lack of certainties or of knowledge? Part 2. Misconceptions, doubts and critical aspects when using generic drugs in the real world. Ital J Med.

[8] Bate R, Jin GZ, Mathur A (2011). Does price reveal poor-quality drugs? Evidence from 17 countries. J Health Econ.

[9] Sheppard A. Generic medicines: essential contributors to the long-term health of society. IMS health. http://www.imshealth.com/imshealth/Global/Content/Document/Market_Measurement_TL/Generic_Medicines_GA.pdf. Accessed 27 Jan 2015.

[10] Gorokhovich LE, Chalkidou K, Shankar R. Improving access to innovative medicines in emerging markets: evidence and diplomacy as alternatives to the unsustainable status quo. J Health Dipl. 2013;1(1).

[11] Shargel L, Isadore K, editors. Generic drug product development: solid oral dosage forms. CRC Press; 2013.

[12] Shah US. Regulatory strategies and lessons in the development of biosimilars. In: Pharmaceutical sciences encyclopedia. Wiley; 2010. doi:10.1002/9780470571224.pse511.

[13] Mastan S, Latha TB, Ajay S. The basic regulatory considerations and prospects for conducting bioavailability/bioequivalence (BA/BE) studies-an overview. Comp Eff Res. 2011;1:1–25.

[14] Kanavos Panos, Costa-Font Joan, Seeley Elizabeth (2008). Competition in off-patent drug markets: issues, regulation and evidence. Econ Policy.

[15] Grabowski HG. Chapter 8: competition between generic and branded drugs. Pharmaceutical innovation: incentives, competition, and cost-benefit analysis in international perspective. 2007, p. 153–288. http://hdl.handle.net/10161/7370.

[16] World Population Growth. World Bank Report. http://www.worldbank.org/depweb/beyond/beyondco/beg_03.pdf. Accessed 27 Jan 2015.

[17] Seiter A. A practical approach to pharmaceutical policy. World Bank Publications; 2010.

[18] Vacca González CP, Fitzgerald JF, Bermúdez JAZ (2006). Definición de medicamento genérico ¿un fin o un medio? Análisis de la regulación en 14 países de la Región de las Américas. Rev Panam Salud Publica..

[19] Vacca C, Claudia V, Martín C, Ludovic R (2011). Publicidad y promoción de medicamentos: regulaciones y grado de acatamiento en cinco países de América Latina; Drug advertising and promotion: regulations and extent of compliance in five Latin American countries. Rev panam salud pública.

[20] WHO National Medicines List/Formulary/Standard Treatment Guidelines. http://www.who.int/selection_medicines/country_lists/en/#K. Accessed 27 Jan 2015.

[21] Nguyen TA, Hassali MA, McLachlan A (2013). Generic medicines policies in the Asia Pacific region: ways forward. WHO South-East Asia J Public Health.

[22] Vogler S (2012). The impact of pharmaceutical pricing and reimbursement policies on generics uptake: implementation of policy options on generics in 29 European countries–an overview. Generics Biosimilars Initiat J.

[23] Shukla N, Sangal T (2009). Generic drug industry in India: the counterfeit spin. J Intellect Propierty Rights..

